# MicroRNA 200a as a histologically independent marker for meningioma recurrence: Results of a four microRNA panel analysis in meningiomas

**DOI:** 10.1002/cam4.5566

**Published:** 2022-12-30

**Authors:** Steffi Urbschat, Benjamin Landau, Nina‐Christin Bewersdorf, Celine Schuster, Gudrun Wagenpfeil, Walter J. Schulz‐Schaeffer, Joachim Oertel, Ralf Ketter

**Affiliations:** ^1^ Department of Neurosurgery Saarland University Medical Center and Saarland University Homburg Germany; ^2^ Institute for Biometrics, Saarland University Homburg Germany; ^3^ Institute of Neuropathology, Saarland University Homburg Germany

**Keywords:** biomarker, chromosome 1p, meningioma, microRNA 200a, recurrence

## Abstract

**Introduction:**

Meningiomas are mostly benign neoplasms of the central nervous system. Nevertheless there are recurrences in about 20% after surgical resection. Previous studies could reveal several predictors of meningioma recurrence. Tumor progression often is associated with a specific pattern of chromosome losses. Our study investigated the potential function of selected microRNAs as markers of tumor progression.

**Methods:**

By real‐time polymerase chain reaction the expressions of microRNA 21‐3p, 34a‐3p, 200a‐3p, and 409‐3p were analyzed in solid tumor and in blood samples of 51 meningioma patients as well as in blood samples of 20 healthy individuals. Additionally, aberrations of parts of chromosomes 1, 14, 18, and 22 were analyzed by FISH. Tumor and blood samples were statistically analyzed, using Spearman's rank correlation coefficient as well as Mann–Whitney U‐ and Kruskal–Wallis‐Test.

**Results:**

MicroRNA 200a showed significantly lower expressions in recurrent meningiomas than in newly diagnosed ones. MicroRNA 409 in meningiomas was correlated significantly with tumor volume and showed a significant negative correlation with patient age. Significance was found between the expression patterns of microRNAs 34a and 200a with the respective aberrations of chromosome 1p and the microRNA 409 with aberration of chromosome 14. In the male cohort the expression of microRNA 200a in blood was significantly upregulated in patients compared to healthy volunteers. By our research the function of microRNA 200a was proved to detect meningioma patients by liquid biopsy.

**Conclusion:**

We detected microRNA 200a as a new biomarker to indicate meningioma recurrences. Future transferability to blood could be important for patient follow‐up.

## Graphical abstract

Workflow process: Comparison of expression levels of microRNAs with chromosomal aberrations and clinical data.

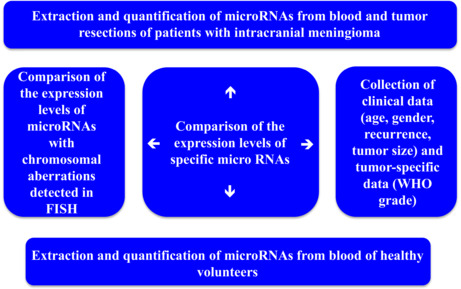

## 1 INTRODUCTION

With approximately one‐third of all primary intracranial brain tumors,^1^ meningiomas are the most common primary intracranial and spinal tumors.^2^, ^3^ The age‐standardized incidence rate per year is higher in woman than in men.^1^, ^4^

Based on the tumor classification of the World Health Organization (WHO), meningiomas are classified into three different histological grades (WHO grades I–III). The benign WHO grade I occurs most frequently.^3^

Nevertheless 20% of the surgical patients develop recurrences after resection.^2^ Since Harvey Cushing's notion concerning the unpredictable course of meningiomas after total resection, various researchers have tried to find prognostic factors for long time results of meningioma surgery. By cytogenetical approach^5^ meningioma was the first solid tumor in which a chromosomal deletion was identified with partial or complete loss of chromosome 22.^6^, ^7^

Using oncogenetic tree mixtures, Ketter et al. showed that cytogenetic evolution of meningiomas is associated with nonrandom loss of additional chromosomes or chromosomal segments.^8^

Based on this Ketter et al. developed a genetic progression score (GPS) which allows to predict meningioma recurrence in a significant way.^5^, ^8^

In this score, aberrations of chromosome 22 and short arm losses of chromosome 1 (1p deletion) play an important role.^5^, ^8^ Chromosome 1p deletion is crucial in malignant progression of meningiomas.^9^ It is associated with significantly worse prognosis and a recurrence rate of 30%.^5^, ^8^, ^10^, ^11^

After monosomy 22 and 1p deletion, in cytogenetic evolution of meningiomas monosomy 14 is found with significantly higher risk of earlier recurrence.^8^

Aberrations of chromosome 18 also play a role in meningioma progression. Monosomy of chromosome 18 is showing later than the previous described aberrations.^8^

MicroRNAs play an important role in RNA interference. By this epigenetic process expression of target genes can be reduced or stopped with high specificity.^12^ Short nonprotein‐coding RNA molecules (silencing RNAs) attach to transcribed messenger RNAs (mRNAs), thus inhibiting gene expression posttranscriptionally.^13^, ^14^

More than 50% of the microRNAs known to date are localized at chromosomal segments containing “fragile sites”. These regions are often genetically altered in tumors.^15^

Preliminary studies have shown that microRNAs show dysregulation in solid meningioma compared to healthy arachnoid cell tissue.^16^

Considering oncogenetic tree mixtures as biostatistical models of clonal cytogenetic evolution, microRNAs localized at chromosomes that are important concerning the recurrence behavior of meningiomas are of particular interest.^8^, ^17^

MicroRNA 21 localized at chromosomal segment 17q23.1 was shown to be upregulated in solid meningioma.^1^, ^18^ Tumors with alterations of chromosomal region 17q may be associated with more infiltrative tumor growth and increased recurrence rate compared to tumors with normal karyotype.^17^ MicroRNA 21 was significantly more highly expressed in WHO grade II and III meningiomas than in WHO grade I tumors.^18^

MicroRNA 409‐3p is localized at chromosomal segment 14q32.31. Monosomy 14 in tumor cells increases the risk of developing atypical and anaplastic meningiomas as well as progression from typical to atypical meningiomas.^8^

MicroRNAs 34a‐3p and 200a are downregulated in solid meningioma compared with healthy arachnoid cell tissue.^1^, ^16^, ^19^, ^20^ MicroRNA 34a‐3p is localized at chromosome 1p36.22 and microRNA 200a is localized at 1p36.33. In Ketter et al. previous' study about oncogenetic tree mixtures in clonal cytogenetic evolution of meningiomas all anaplastic meningiomas showed deletion of chromosome 1p.^8^

Higher histopathological grade of meningiomas is associated with lower expression level of microRNA 34a‐3p.^16^, ^19^

MicroRNA 200a has a tumor suppressive effect and is greatly reduced in meningioma cells. In consequence expression of ß‐catenin and cyclin D1 is increased. These proteins take part in regulation of cell proliferation.^20^

The described investigations on meningiomas have three objectives:

First of all, the expression of microRNAs in blood of meningioma patients are compared to blood of healthy controls. The qPCR‐based microRNA analysis is validated by an endogenous blood control of meningioma patients.

We analyze whether expression levels of microRNAs differ between initial diagnoses and recurrences in the same way as the respective chromosomes. Additionally microRNA levels are analyzed concerning several clinical variables.

Furthermore we analyze the correlation of microRNA expression patterns between tumor and blood.

Finally, the expression patterns of microRNAs in meningiomas are compared to chromosomal aberrations detected in fluorescence in situ hybridization (FISH).

## 2 MATERIAL AND METHODS

### 2.1 Patient population

The study was approved by the institutional review board (IRB) of the medical board of Saarland (number 02/20). Written informed consent was obtained from each person participating in the study.

We enrolled a prospective study on 51 patients who underwent meningioma surgery at the Department of Neurosurgery, Saarland University between June 2019 and August 2020.

Statistics were performed based on the validation of RNU6B as endogenous control in blood samples of 43 patients (32 females; 11 males). In this cohort 36 newly diagnosed meningiomas were compared to seven tumor recurrences.

Mean age of the entire patient population was 61.7 years (standard deviation: 13.85 years), mean age of female patients was 61.5 years, and mean age of male patients was 62.4 years.

Additionally, blood of 20 healthy control volunteers (12 females; eight males) was taken in order to compare microRNA expression levels.

Mean age of the healthy control volunteers was 36 years (standard deviation: 15 years), with mean age of 34 years in female volunteers, and mean age of 40 years in male group.

### 2.2 Clinical variables

The clinical variables comprise patient sex and age, tumor size, histology (WHO grade), and newly diagnosed meningioma versus recurrence.

### 2.3 Tumor histology

The 43 meningiomas included 35 of common type (WHO grade I), seven of intermediate type (WHO grade II) and one anaplastic meningioma (WHO grade III). Histologic subtypes were defined according to World Health Organization (WHO) criteria and diagnosed by one of the authors.

### 2.4 Patient samples

After receiving written informed patient consent, tumor and blood samples were prospectively obtained from patients undergoing treatment at the Department of Neurosurgery, Saarland University.

After tumor resection, the tissue was stored at −80°C until analysis.

Immediately before surgery, up to 5 ml of blood was drawn from each patient. The same amount of blood was drawn from healthy control volunteers. Samples were centrifuged immediately afterward at 1900*g* for 10 min at 4°C. Then plasma was transferred to a fresh tube and centrifuged again at 16000*g* for 10 min at 4°C. The sample was stored at −20°C until analysis. The blood of healthy volunteers was processed in the same way. Follow‐up data were obtained from the patients' medical records.

### 2.5 Serum RNA purification and quantification

Total RNA from tumor samples was extracted using the Qiagen miRNeasy® Mini Kit according to manufacturer's instructions.

Total RNA from plasma samples of patients and healthy controls was extracted using Qiagen miRNeasy® Serum/Plasma Kit. Based on manufacturers instructions, twice the amount of blood plasma was used to achieve the required RNA concentration of 17–23 ng/μl.

Eluted RNA from tumor tissue and plasma samples was quantified using a nano‐drop spectrophotometer (Thermo Fisher Scientific). The RNA obtained from all tumor tissue samples had to be diluted to a concentration of 23 ng/μl.

### 2.6 Reverse transcription polymerase chain reaction

Reverse transcription polymerase chain reaction was applied for transcribing RNA into complementary DNA (cDNA).^21^ cDNA was used for subsequent real‐time polymerase chain reaction (qPCR) to measure the amount of a specific RNA. RNU6B and RNU48 were used in order to detect a new endogenous control for qPCR based microRNA analysis in blood of meningioma patients.

For each examined pair of microRNA/RNU and patient sample, the general master mix was prepared.

The master mix was composed of a fixed amount of 0.15 μl dNTP Mix, 1.5 μl RT buffer, 0.19 μl RNase inhibitor, 1 μl reverse transcriptase (TaqMan™ MicroRNA Reverse Transcription Kit), 4.16 μl PCR water (Invitrogen by Thermo Fisher Scientific), and 3 μl of the respective RT primer (Applied Biosystems™ by Thermo Fisher Scientific) for each examined pair of microRNA/RNU and patient sample. Five micro liter of the RNA elute was added.

Then the samples were placed in a thermal cycler. The procedure was as follows: 30 min at 16°C, 30 min at 42°C, and 5 min at 85°C. Subsequently, the samples were stored at 4°C for an hour until real‐time quantitative PCR.

### 2.7 Real‐time quantitative PCR (qPCR)

The qPCR was performed using TaqMan® MicroRNA Assays. TaqMan® MicroRNA Assays consist of primers and TaqMan hydrolysis probes.

After enzyme activation at 95°C for 10 min, denaturation (15 s) as well as annealing and extension (1 min) steps were repeated alternately 45 times. All reactions were performed in triplicate. Each reaction was composed of 0.5 μl TM primer (Applied Biosystems™ by Thermo Fisher Scientific), 5 μl TaqMan™ Gene Expression Master Mix (Applied Biosystems™ by Thermo Fisher Scientific), and 3.5 μl PCR water (Invitrogen by Thermo Fisher Scientific). Then 1 μl of the respective cDNA was added per reaction. A water control was used for each analyzed RNA/RNU.

Experiment and measurement were performed in a StepOnePlusTM Real‐Time PCR System with the corresponding StepOne Software v2.3.

### 2.8 Data normalization by validation of RNU6B in plasma

The results of qPCR‐based microRNA analysis are influenced by several factors. Sample collection, further processing, storage and efficiency of enzymes alter the measured values of the analysis.^22^, ^23^ Therefore accurate data normalization is required. In relative quantification the target microRNA is compared with a stably expressed endogenous control from the same sample (“housekeeping transcript”).^22^ By using an endogenous control, it is possible to eliminate factors influencing the quality of RNA. Sampling differences (in quantity and quality of RNA) can also be computationally leveled. This is because both microRNA and endogenous control are affected by all variables that might influence the analysis^22^, ^23^

A universally accepted reference gene for the analysis of all microRNAs does not exist.^22^ Consequently, an endogenous control must be properly validated.^23^ Among the commonly used endogenous controls RNU6B and RNU48^16^, ^20^, ^22^ we aim to validate a new reference gene for qPCR based microRNA analysis in blood of meningioma patients. To date, no microRNA expression analysis has been performed in the blood of meningioma patients based on an endogenous control. Cut‐off for successful validation was set at standard deviation of one around the mean. The expression of RNU48 was unstable. RNU6B Ct value standard deviation less than one was applicable for 43 of the 51 analyzed tumors. Consequently, the expression levels of the microRNAs 21, 34a, 200a, and 409 were normalized to RNU6B. This was applicable for 43 of the 51 analyzed tumors. Samples not falling within this range had obviously been stored for too long, therefore RNU6B expression was unstable.

### 2.9 Statistical Analysis

Statistical analysis was based on relative expression to evaluate expression patterns in microRNAs.^24^ SPSS version 25 was used to perform statistical analysis by applying Spearman's rank correlation coefficient as well as Mann–Whitney U‐ and Kruskal–Wallis‐Test.

$\text{Relative Expression}:2^{-\left(\text{∆}\;\mathrm{Ct}\right)}=2^{-\left(\mathrm{Ct}\;\left(\text{microRNA}\right)—\mathrm{Ct}\;\left(\mathrm{RNU}6B\right)\right)}$

### 2.10 Fluorescence in situ hybridization (FISH)

For the analysis of aberrations of chromosome 1, 14, 18, and 22, fluorescence in situ hybridization (FISH) was performed in native tumor tissue (dapped slides).

Two‐color hybridization DNA‐probes were used to detect the chromosomal regions 1p36/22q11 and 14q24/18q23 (MetaSystems GmbH). The procedure has been performed in previous studies in our laboratory.^17^, ^25^ Signals were analyzed with an Olympus BX43 fluorescence microscope based on the criteria of Hopman et al.^26^ Two hundred cell nuclei per pair of hybridization probes were counted with a cut‐off value of 6%.

## 3 RESULTS

### 3.1 Healthy volunteers

**FIGURE 1:**
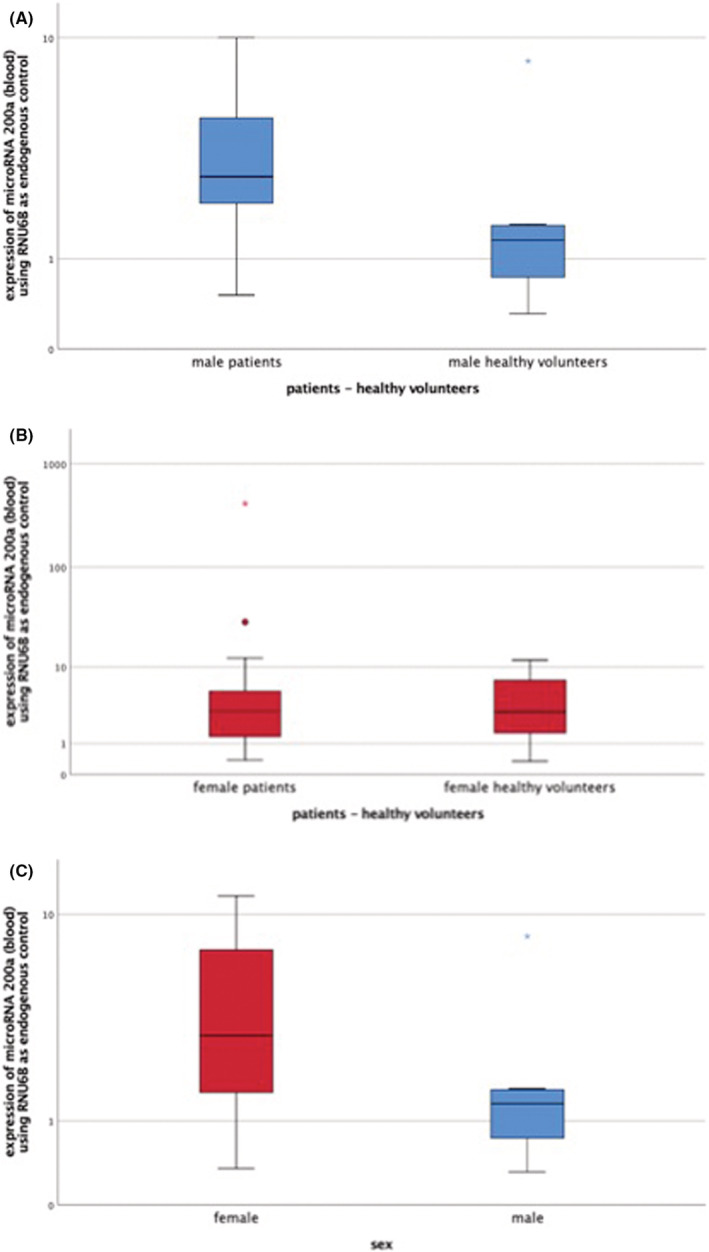
Expression patterns of microRNA 200a in blood of healthy volunteers and patients; gender specific comparison in healthy volunteers. (A) Comparison of microRNA 200a expression patterns between male patients and healthy male volunteers (*p* = 0.016). (B) Comparison of microRNA 200a expression patterns between female patients and healthy female volunteers (*p* = 0.805). (C) Comparison of microRNA 200a expression patterns between healthy female and male volunteers (*p* = 0.057).

Table 1 shows that comparison between blood from 43 meningioma patients and 20 healthy volunteers revealed significant difference concerning microRNA 200a. In male patients, this marker was significantly upregulated compared to healthy male volunteers (Figure 1A). This difference was not found in the female cohort (Figure 1B).

**TABLE 1:** Expression pattern of microRNAs 21, 34a, 200a, and 409 in blood of healthy volunteers and patients with a gender specific comparison in healthy volunteers

	miR 21	miR 34a	miR 200a	miR 409
Healthy controls versus meningioma patients (females and males)	0.114	0.434	0.256	0.565
Healthy controls versus meningioma patients (female cohort)	0.153	0.630	0.805	0.687
Healthy controls versus meningioma patients (male cohort)	0.442	0.545	**0.016**	0.840
Healthy controls: female cohort versus male cohort	0.521	0.427	0.057	0.624

In a gender specific differentiation in the healthy cohort, microRNA 200a was shown to be more highly expressed in the blood of women than in men (Figure 1C). The significance level of 0.05 was only narrowly missed.

Concerning microRNAs 21, 34a, and 409 there was no significant expression difference between meningioma patients and healthy volunteers.

### 3.2 Meningioma patients

After successful validation of RNU6B as endogenous control in blood, we analyzed tumor and blood of 43 meningioma patients.

Table 2 compares expression patterns of microRNAs 21, 34a, 200a, and 409 between solid tumor and blood and presents an analysis concerning clinical parameters.

**TABLE 2:** Expression pattern of microRNAs 21, 34a, 200a, and 409 in solid tumor (T) and in blood (B) concerning the analyzed clinical parameters

	miR 21 (T)	miR 34a (T)	miR 200a (T)	miR 409 (T)
Age groups (<60 years; ≥60 years)	0.865	0.716	0.224	**0.046**
Newly diagnosed meningiomas versus recurrences	0.860	0.187	**0.009**	0.223
Sex (female; male)	0.816	0.794	0.671	0.483
Tumor volume groups (≤15 cm^3^; >15 cm^3^–40 cm^3^; >40 cm^3^)	0.900	0.475	0.667	**0.031**
WHO grade I versus WHO grade II	0.692	0.099	0.257	0.792

	miR 21 (B)	miR 34a (B)	miR 200a (B)	miR 409 (B)
Age groups (< 60 years; ≥ 60 years)	0.466	0.319	0.528	0.981
Newly diagnosed meningiomas versus recurrences	0.410	0.097	0.410	0.962
Sex (female; male)	0.276	0.450	0.816	0.501
Tumor volume groups (≤15 cm3; >15 cm3–40 cm3; >40 cm3)	0.770	0.255	0.749	0.402
WHO grade I versus WHO grade II	0.508	0.446	0.487	0.257

Bold indicates statistical significant value (*p* ≤ 0.05).

Table 3 focuses on several clinical parameters concerning aberrations of chromosomes 1, 14, 18, and 22 in meningiomas and compares expression patterns of microRNAs with respective results concerning chromosomal aberrations in FISH.

**TABLE 3:** Aberrations of chromosomes 1, 14, 18, and 22 in the analyzed solid meningiomas concerning selected clinical parameters and comparison of the expression patterns of the microRNAs with the respective results concerning chromosomal aberrations in FISH

	chromosome 1	chromosome 14	chromosome 18	chromosome 22
Newly diagnosed meningiomas versus recurrences	**0.013**	0.102	0.237	0.900
Tumor volume groups (≤15 cm^3^; >15 cm^3^–40 cm^3^; >40 cm^3^)	0.872	0.074	0.358	0.160
Age (<60 years; ≥60 years)	0.987	0.277	0.497	0.776

	miR 34a—chromosome 1	miR 200a—chromosome 1	miR 409—chromosome 14
microRNA—chromosome	**≤ 0.001**	**≤ 0.001**	**≤ 0.001**

Bold indicates statistical significant value (*p* ≤ 0.05).

Comparing tumor and blood samples of the individual meningioma patients, no significant correlation was obtained concerning the expression levels for any of the analyzed microRNAs.

Concerning clinical parameters, sex and histology (WHO grade), no significant difference could be detected in tumor versus blood of meningioma patients.

**FIGURE 2:**
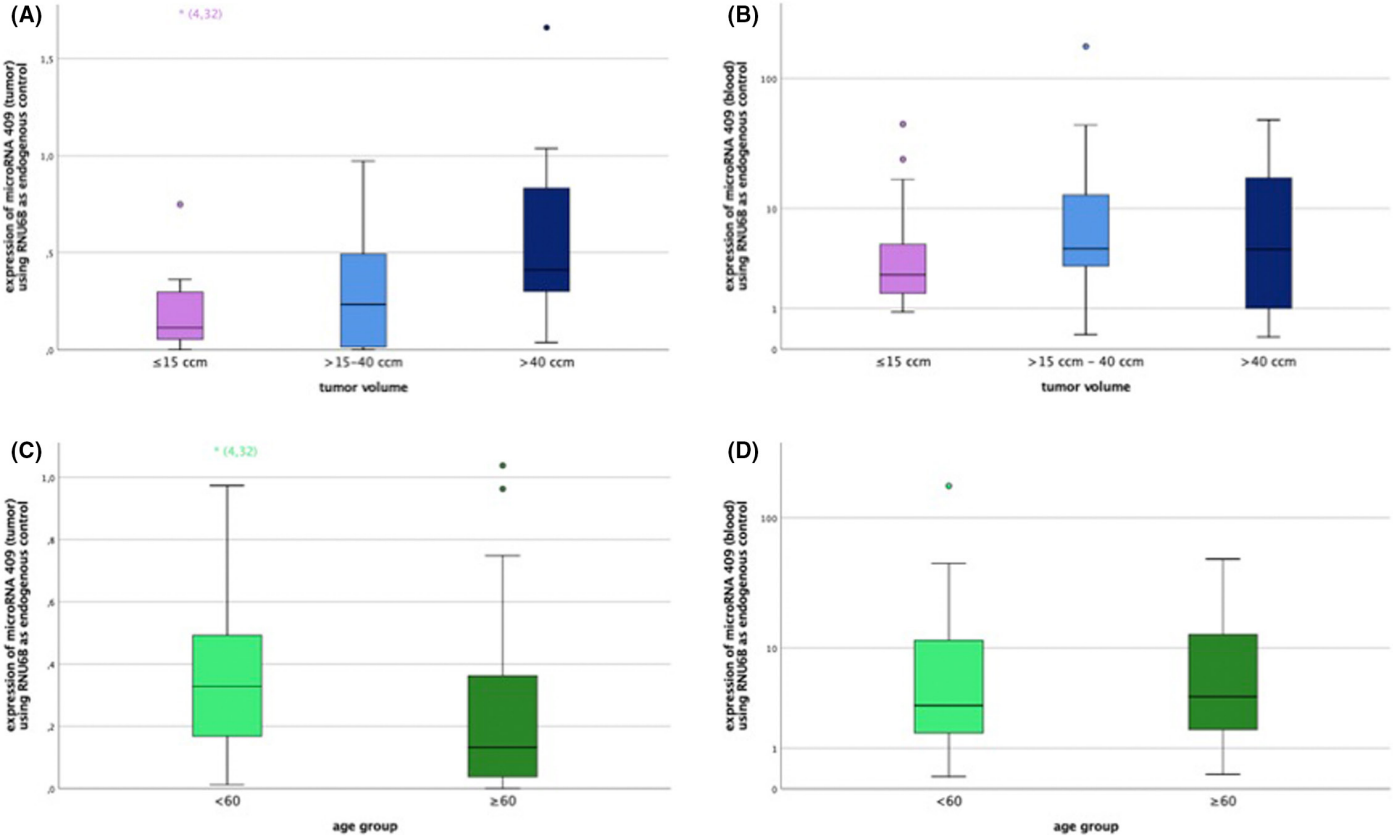
Expression patterns of microRNA 409 according to groups of tumor volumes and age of patients. (A) Expression patterns of microRNA 409 according to three ascending groups of tumor volumes in solid meningioma (*p* = 0.031) and (B) in blood (*p* = 0.402) (Group 1: ≤ 15 cm^3^; Group 2: >15 cm^3^—40 cm^3^; Group 3: > 40 cm^3^). (C) Expression patterns of microRNA 409 according to two age groups in solid meningioma (*p* = 0.046) and (D) in blood (*p* = 0.981) (Group 1: < 60 years; Group 2: ≥ 60 years).

With regard on tumor size, tumor volumes were divided into three ascending groups (≤15 cm^3^, >15 cm^3^–40 cm^3^, and >40 cm^3^). Figure 2A shows that expression levels of microRNA 409 significantly increased with the size of tumor volume group (*p* = 0.031). This was not the case in blood analysis (*p* = 0.402) (Figure 2B). Figure 2C,D displays the differences of the microRNA 409 expression patterns concerning two age groups. The microRNA 409 level in tumors of patients at the age of 60 or older was significantly lower than in patients younger than 60 years (*p* = 0.046) (Figure 2C). This result could not be found in blood (*p* = 0.981) (Figure 2D).

**FIGURE 3:**
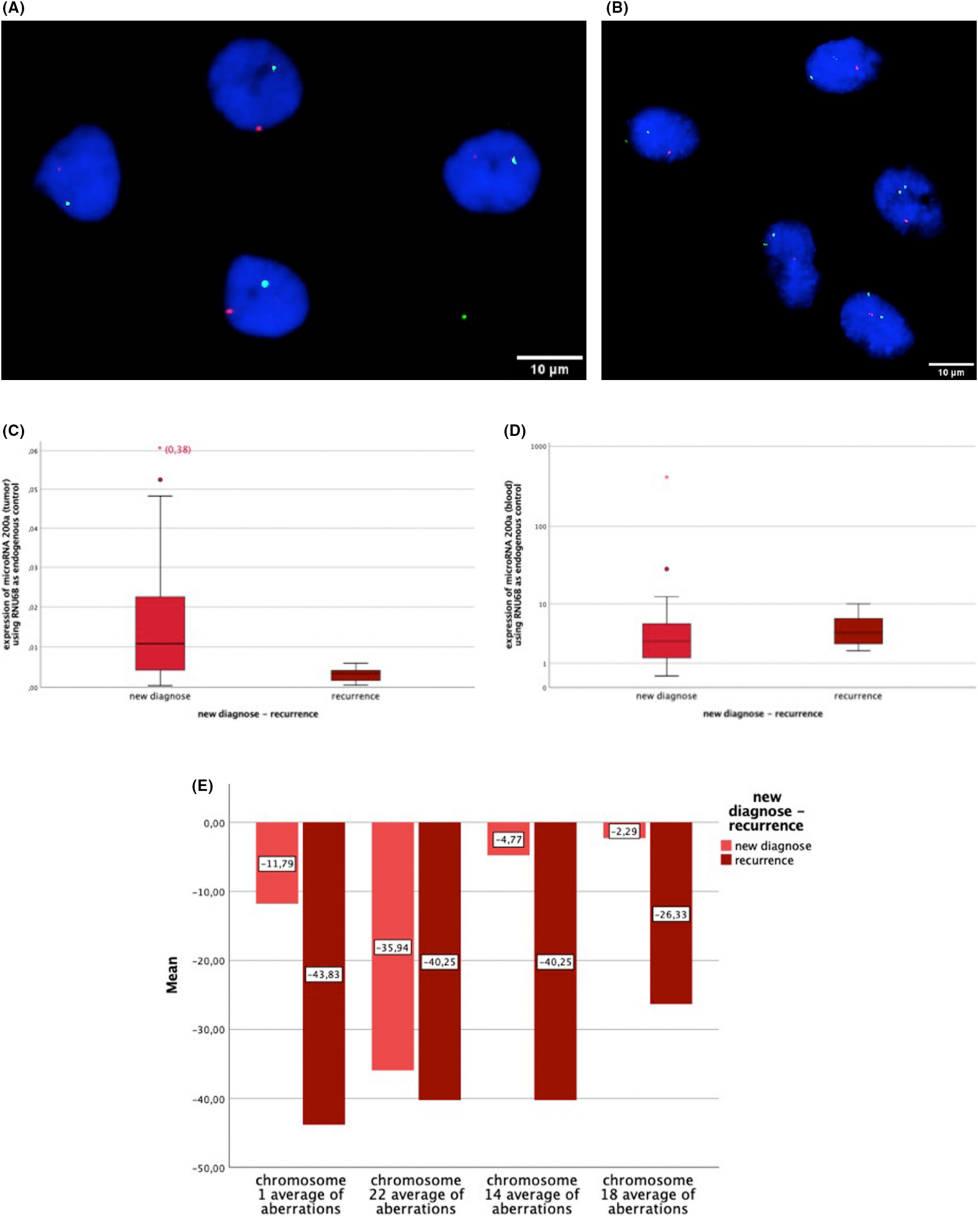
Expression patterns of microRNAs and of chromosomes of newly diagnosed meningiomas and recurrences. (A) FISH analysis of meningioma cells with two‐color hybridization probes for the identification of chromosome region 1p36 (red spot)/22q11 (green spot). Four cell nuclei (DAPI stained in blue), with deletions of chromosome 1p and 22q (one red and one green signal each). (B) FISH analysis of meningioma cells with two‐color hybridization probes for the identification of chromosome region 14q24 (red spot)/18q21 (green spot). Five cell nuclei (DAPI stained in blue), with a deletion of chromosome 14q and a diploid 18q chromosome set (one red signal and two green signals). (C) Expression patterns of microRNA 200a in solid meningioma (*p* = 0.009) and (D) in blood (*p* = 0.410). (E) Average of aberrations related to 200 cell nuclei of chromosome 1p (*p* = 0.013), 22 (*p* = 0.900), 14 (*p* = 0.102), and 18 (*p* = 0.237) in newly diagnosed meningiomas and recurrences in percent. A significant difference could be obtained concerning chromosome 1p. The “cut‐off value” was 6% related to one sample.

Analyzing the difference in microRNA expression between newly diagnosed meningiomas and recurrences, a significant difference was evident concerning microRNA 200a. Tumor microRNA 200a was significantly less expressed in recurrences than in newly diagnosed meningiomas (*p* = 0.009) (Figure 3C). This was not the case in blood (*p* = 0.410) (Figure 3D).

With regard to the other analyzed microRNAs, no significant difference could be detected concerning newly diagnosed meningiomas and tumor recurrences.

A related analysis was also performed on chromosomes that play a role in the recurrence behavior of meningiomas (Figure 3E).

Based on the quantification of chromosomal aberrations in percent, a comparison between newly diagnosed tumors and recurrences showed significantly more losses of chromosome 1p in recurrences. With regard to the other analyzed chromosomes 14, 18, and 22, no significant difference was detectable.

A comparison of the expression patterns of the microRNAs with the respective results in FISH (microRNA 34a and 200a—chromosome 1p; microRNA 409—chromosome 14) was highly significant (p ≤ 0.001).

The median age of patients with newly diagnosed meningiomas was 60 years versus 70 years for tumor recurrences.

## 4 DISCUSSION

In this study we analyzed the role of microRNAs 21, 34a, 200a, and 409 in meningiomas. A special focus was the distinction of healthy volunteers and meningioma patients by liquid biopsy.

For our prospective experimental study, tumor samples of newly incoming clinical patients were needed. Nevertheless with 43 cases the number was adequate for statistical analysis. Thus the highly significant outcome of our main results are robust and reliable. Data from in silico analyses were not needed and could not be used for this very unique experimental setup. Indeed it was the very first time, that an endogenous control, RNU 6B, was used for the analysis of microRNA expression in blood, which is the gold standard of microRNA expression controls.

MicroRNA 200a was more highly expressed in plasma of male meningioma patients compared to healthy males. Basically this difference could be caused by the higher number of cells in tumors compared to healthy tissue and consequently the increased release of the respective microRNA into blood. Hypothesis for the sex differences are described below.

It was shown that microRNA 200a was more highly expressed in the blood of female healthy volunteers than in males. This difference was just barely not significant. Comparison of the other microRNAs showed no gender‐specific difference.

MicroRNAs control the expression of target genes via RNA interference. Their regulation is effected by various signaling cascades.^12^, ^19^, ^27^ Marton et al. showed the influence of hormones concerning these pathways. Estradiol has a regulatory function on a variety of microRNAs. The ligand for estradiol is estrogen receptor alpha (ER α). In an ovarian cell line expressing ER α, higher expression of microRNA 200a was detected than in cells which did not express this receptor.^27^ This could be the reason why microRNA 200a is more highly expressed in females than in males. MicroRNA 200a could be masked in the blood of women with meningioma by the naturally increased microRNA 200a expression. Although microRNA 200a is not specific for meningiomas, increased expression of microRNA 200a in blood plasma of male meningioma patients could lead toward a blood‐based test to detect meningiomas.

In this trial, we mainly focused on the different expression patterns between newly diagnosed meningiomas and tumor recurrences.

We revealed that microRNA 200a localized at chromosome 1p was significantly lower expressed in recurrences than in newly diagnosed meningiomas. With regard to chromosome 1p, this result could be confirmed. No expression difference of microRNA 200a was detected between WHO grades.

Loss of chromosome 1p is considered the most significant recurrence marker for meningiomas.^5^, ^10^, ^11^, ^28^ In tumor progression, this marker is independent from the histological classification.^8^

MicroRNA 200a affects meningioma growth in vivo and in vitro. Compared to healthy arachnoid cell tissue microRNA 200a in WHO grade I meningiomas showed a decrease by approximately 25‐fold.^20^ MicroRNA 200a has a critical influence on the regulatory proteins ß‐catenin and cyclin D. WNT/ß‐catenin signaling pathway moreover is relevant in various tumor diseases such as recurrence behavior of colon carcinomas.^20^ MicroRNA 200a has a direct inhibitory effect on translation of ß‐catenin mRNA and thus on the WNT/ß‐catenin signaling pathway.^20^ In meningioma a direct correlation between downregulation of microRNA 200a and upregulation of ß‐catenin could be shown.^20^ Furthermore, downregulation of microRNA 200a in meningiomas decreased expression of the cellular adhesion protein E‐cadherin which influences cell differentiation. In malignant tumors, reduction of cadherins often plays a role. In addition, decreased levels of E‐cadherin increase the availability of ß‐catenin in the cytoplasm or nucleus. Thus downregulation of microRNA 200a has an impact on carcinogenesis via two converging pathways. Saydam et al. showed.

in cell culture and in a xenograft tumor model in vivo that artificially upregulated levels of microRNA 200a in meningiomas reduce tumor growth. Thus, microRNA 200a acts as a tumor suppressor in meningiomas.^20^ This has been confirmed in other tumor studies, example downregulation of microRNA 200a promotes cell proliferation in human glioma.^29^ Its overexpression in gliomas improves sensitivity of temozolomide responsiveness.^30^

Meningioma recurrences are often defined by more aggressive growth than initial diagnoses.^3^, ^5^, ^10^, ^11^ Therefore downregulation of the investigated microRNA 200a may be associated with meningioma recurrences.

Regarding the analyzed tumors, a highly significant correlation could be found between the expression patterns of microRNAs 34a and 200a with the respective results of chromosome 1p in FISH. Comparison of microRNA 409 with chromosome 14 had the same outcome.

In 2018 Dürrbaum et al. showed for the first time, that the expression of microRNAs can be altered in response to chromosomal gain.^31^ In our study, we could confirm these preliminary results and specified them by analyzing 200 cell nuclei: In meningiomas the number of chromosomal deletions or gains has a highly significant effect on the expression of the respective microRNA.

This indicates that the amount of corresponding microRNA expression in tumor is caused by the extend of respective chromosomal aberrations. Thus comparability between the molecular cytogenetic and the epigenetic level is proved.

Although microRNA 34a is located on the short arm of chromosome 1 it was not significantly downregulated in recurrent tumors. A possible explanation is less frequent deletion of the genome sequence encoding microRNA 34a (1p36.22) than the region of microRNA 200a (1p36.33) in meningioma recurrences.

Based on the discovery of microRNA 200a as a WHO grade independent progression marker in meningioma it would be of translational relevance to transfer the detection of this marker into blood. Implementation of a blood‐based biomarker could be a cost‐effective and simply applicable way of patient follow‐up. Furthermore, a potential therapeutic overexpression of the tumor‐suppressive microRNA 200a in meningioma recurrences and in newly diagnosed meningiomas might be a therapeutic approach to reduce or even stop tumor growth.

Concerning chromosome 22 no difference was detected between newly diagnosed meningiomas and recurrences. This is compatible with our previous finding that monosomy 22 is not relevant for prognosis.^5^ Chromosome 14 and 18 were less expressed in recurrences than in newly diagnosed meningiomas although no significance was reached. Monosomy 14 and 18 occur later in cytogenetic evolution of meningiomas than deletion of chromosome 1p. This confirms the important role of chromosome 1p as an earlier prognostic marker for meningioma recurrence.^5^, ^10^, ^11^, ^28^

Although the majority of microRNAs are located in the intracellular compartment former studies showed that they can be detected in body fluids. Several explanations are possible: Besides of active secretion of microRNAs due to special stimuli or via cell‐derived microvesicles microRNAs can also leak into body fluids by cell disruption.^32^

The transport of molecules into or out of the central nervous system is modulated by the blood–brain barrier.^33^ Meninges however are not part of this physiological barrier.^34^ Because the blood–brain barrier is not affected in meningioma patients, the secretion of microRNAs into blood might not necessarily be significantly increased. Probably for this reason, the expression patterns of the analyzed microRNAs do not correlate between tumor and blood.

Concerning the analyzed microRNAs 21, 34a, 200a, and 409 in tumor tissue a significant correlation with tumor volume could only be shown for microRNA 409. This indicates an increase of microRNA 409 expression while total RNA concentration of 23 ng/μl remained constant.

This result may be due to the fact that progression of meningioma is often associated with hypodiploidy of certain chromosomes.^8^ However in some rare cases hyperdiploidy occurs with a tendency to higher histological grades.^8^, ^35^ Interestingly, chromosome 1 (microRNAs 34a and 200a) is not affected by hyperdiploidy.^35^ Chromosome 14 (microRNA 409) and chromosome 17 (microRNA 21) sometimes exhibit hyperdiploid chromosomes.^35^ In this trial, chromosome 14 expression differences were also examined by FISH with respect to the three groups of ascending tumor sizes. Significance was just narrowly missed. In this context, it could be shown that compared to smaller tumor sizes the group of the largest meningiomas (>40 cm^3^) was more likely to be associated with hyperdiploidies. Since the expression of microRNAs correlates highly significant with chromosomal aberrations we assume that larger meningiomas with a tendency to hyperdiploidy in chromosome 14 are also responsible for an increase in microRNA 409. In smaller meningiomas, we could show that hypodiploidy predominates. This could be the reason why only microRNA 409 can be correlated significantly with tumor volume.

In a future project, it would be interesting to find out whether larger meningiomas can be significantly associated with hyperdiploidy compared to smaller ones.

Regarding differences in microRNA expression patterns with respect to patient age, microRNA 409 in solid tumor was the only microRNA that showed a significant negative correlation with patient age. The cohort of patients 60 years and older showed a significantly lower expression of microRNA 409 than the younger patient group.

Chromosome 14 was also found to be more often affected by losses in patients of 60 years and older than other chromosomes of this investigation. However no significance was obtained. Expression of microRNA 409 correlated highly significant with losses of the respective chromosome 14. The reasoning is based on the biostatistical model of clonal cytogenetic evolution of meningiomas.^5^ The median age of patients with newly diagnosed tumors was 60 years versus 70 years for recurrences. Loss of chromosome 14 in clonal cytogenetic evolution is a later event than loss of 1p.^8^ With increasing age of patients the cytogenetic evolution of meningiomas tends to be more advanced and more losses of chromosome 14 are detected. This is a possible reason why microRNA 409 is more highly expressed in the younger cohort of patients than in older ones. Additionally, Noren Hooten et al. showed in a genome‐wide assessment of microRNA expression in peripheral blood mononuclear cells that expression patterns of microRNAs generally decrease with age.^36^

## 5 CONCLUSION

To summarize this study, we detected the microRNA 200a as a new recurrence marker in meningiomas. In a next step a transfer of these results to blood would be beneficial to diagnose meningioma recurrences through a easily manageable liquid biopsy procedure.

Furthermore in the plasma of the male meningioma patients the expression of microRNA 200a was significantly higher than in the control group of healthy males. These results are valid not only for recurrences but for first diagnoses as well and could for the first time lead to a gender specific approach of microRNA 200a as marker of meningiomas by liquid biopsy.

## AUTHOR CONTRIBUTIONS

**Steffi Urbschat:** Conceptualization (lead); funding acquisition (lead); methodology (lead); project administration (lead); resources (lead); supervision (lead); writing – original draft (equal); writing – review and editing (lead). **Benjamin Landau:** Conceptualization (equal); data curation (lead); formal analysis (lead); funding acquisition (supporting); investigation (lead); methodology (lead); project administration (equal); software (supporting); validation (equal); visualization (lead); writing – original draft (lead); writing – review and editing (lead). **Nina‐Christin Bewersdorf:** Data curation (equal); investigation (equal); visualization (equal); writing – original draft (supporting); writing – review and editing (supporting). **Celine Schuster:** Data curation (equal); investigation (equal); visualization (equal). **Gudrun Wagenpfeil:** Formal analysis (supporting); methodology (supporting); software (supporting); supervision (supporting); visualization (equal); writing – review and editing (supporting). **Walter J. Schulz‐Schaeffer:** Investigation (supporting); writing – review and editing (supporting). **Joachim Oertel:** Conceptualization (lead); funding acquisition (lead); project administration (lead); supervision (lead); writing – review and editing (lead). **Ralf Ketter:** Conceptualization (lead); funding acquisition (lead); methodology (lead); project administration (lead); supervision (lead); writing – original draft (equal); writing – review and editing (lead).

## FUNDING INFORMATION

This work was supported by the Saarland University Faculty of Medicine.

## CONFLICTS OF INTEREST

The authors declare that there is no conflict of interest.

## ETHICAL APPROVAL STATEMENT

The study was approved by the institutional review board (IRB) of the medical board of Saarland (number 02/20).

## Data Availability

The data that support the findings of this study are available from the corresponding author upon reasonable request.
